# Dicationic Alkylammonium Bromide Gemini Surfactants. Membrane Perturbation and Skin Irritation

**DOI:** 10.1371/journal.pone.0026965

**Published:** 2011-11-10

**Authors:** João A. S. Almeida, Henrique Faneca, Rui A. Carvalho, Eduardo F. Marques, Alberto A. C. C. Pais

**Affiliations:** 1 Chemistry Department, University of Coimbra, Coimbra, Portugal; 2 Department of Life Sciences, Centre for Neurosciences and Cellular Biology, University of Coimbra, Coimbra, Portugal; 3 Department of Chemistry and Biochemistry, Centro de Investigação em Química, University of Porto, Porto, Portugal; King's College, London, United Kingdom

## Abstract

Dicationic alkylammonium bromide gemini surfactants represent a class of amphiphiles potentially effective as skin permeation enhancers. However, only a limited number of studies has been dedicated to the evaluation of the respective cytotoxicity, and none directed to skin irritation endpoints. Supported on a cell viability study, the cytotoxicity of gemini surfactants of variable tail and spacer length was assessed. For this purpose, keratinocyte cells from human skin (NCTC 2544 cell line), frequently used as a model for skin irritation, were employed. The impact of the different gemini surfactants on the permeability and morphology of model vesicles was additionally investigated by measuring the leakage of calcein fluorescent dye and analyzing the NMR spectra of ^31^P, respectively. Detail on the interaction of gemini molecules with model membranes was also provided by a systematic differential scanning calorimetry (DSC) and molecular dynamics (MD) simulation. An irreversible impact on the viability of the NCTC 2544 cell line was observed for gemini concentrations higher than 25 mM, while no cytotoxicity was found for any of the surfactants in a concentration range up to 10 mM. A higher cytotoxicity was also found for gemini surfactants presenting longer spacer and shorter tails. The same trend was obtained in the calorimetric and permeability studies, with the gemini of longest spacer promoting the highest degree of membrane destabilization. Additional structural and dynamical characterization of the various systems, obtained by ^31^P NMR and MD, provide some insight on the relationship between the architecture of gemini surfactants and the respective perturbation mechanism.

## Introduction

Transdermal drug delivery has been indicated as one of the most promising routes for drug administration. Although the actual barrier function of the skin limits the permeation of drugs, some chemical compounds with the ability to modulate skin properties have been proposed in order to improve skin permeation and achieve therapeutic doses of the drug [1]. However, chemical permeation enhancers (CPE) reduce skin diffusional resistance by reversibly altering the physicochemical properties of the stratum corneum. Therefore, a major consequence is that CPE may cause damage and skin irritation, limiting their usefulness for therapeutical application [2].

Amphiphiles, in general, are known to influence the organization of lipid membranes and, particularly, surfactants have been subjected to intense study in systems involving interaction with lipid membranes [3]. Those of positive charge are typically more effective as permeation enhancers than the alternative anionic and nonionic compounds, although cytotoxicity is potentially more significant in cationic systems [4], [5]. Among several classes of surface-active compounds, dicationic alkylammonium bromide gemini surfactants were chosen, since they are known to efficiently modulate the order in biomembranes as indicated in a previous publication by some of the authors [6].

Gemini surfactants are a class of amphiphiles constituted by two hydrophobic tails and two hydrophilic headgroups covalently connected by a spacer. From a structural perspective, they can be thought of as two single-tail surfactants connected by a spacer that may present variations in terms of hydrophobicity, length and rigidity [7], [8]. In the last two decades, many contributions have been made on the characterization of these materials from a colloidal perspective [9], [10]. The assessment of biological effects [11], [12] and respective interaction with polymers [13] and other relevant biological molecules [14] have also been reported. The simplest and most studied cationic gemini surfactants are the quaternary ammonium compounds [15], represented by the general structure
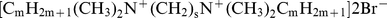



These surfactant molecules, often simplified to 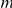-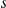-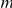, present two identical ammonium head groups connected by a saturated alkyl chain spacer with 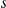 carbons, and two symmetric saturated alkyl tails comprising 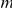 carbons. Interesting aggregation properties have been attributed to these molecules, characterized by very low critical micelle concentration (CMC) values [9], [10], [15], when contrasted to their single-tail counterpart, such as dodecyltrimethylammonium bromide (DTAB) [7].

As far as dicationic alkylammonium bromide gemini surfactants are concerned, only a limited number of studies on the cytotoxicity of such compounds has been published, and none, to our knowledge, has been directed to the effect upon the skin. In a previous study, in which the same type of gemini surfactants (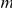-4-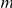, 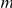 = 8, 11, 13, 16) was assessed on erythrocyte cells [16], the results indicate that gemini disturb human erythrocytes and that the hemolytic potency increases as the alkyl chain length increases. It has also been suggested that 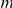-4-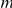 gemini surfactants disturb the membrane in a way similar to single-chain cationic amphiphiles, but that they do not easily translocate to the inner membrane leaflet. Also, in a very recent work conducted by some of the authors, complexes of 14-2-14:DNA and different formulations containing cholesterol (Chol) and Chol:DOPE were assessed in terms of cytoxicity upon TSA cells [17]. In this case, a relatively low toxicity level has been found.

Evaluation of skin irritation has traditionally been conducted in animals. However, beyond the obvious ethical implications, *in vivo* tests present several disadvantages concerning reproducibility and cost. Furthermore, the increasing knowledge of the basic mechanisms of cutaneous inflammation and advanced techniques to cultivate human skin cells led to the recommendation of *ex vivo* approaches as an alternative to animal testing [18]. Skin irritation is a reversible inflammatory reaction produced by the arachidonic acid cascade and cytokines in the viable keratinocytes and fibroblasts of the skin [19]. Therefore, the assessment of potential skin irritation of surfactants resorting to human keratinocytes has been extensively used [19]–[22].

In this work, keratinocyte cells from the human skin (NCTC 2544 cell line) were used as a model for skin irritation studies, while a resazurine test [23] was used to evaluate the cell viability after 24 h of gemini surfactants exposition. The ability of cells to recover after removing the aggression agent was also evaluated 48 h later. Resorting to a systematic DSC study [6] and a fluorescent dye-leakage assay [24], the mechanism of membrane disruption was additionally investigated by inspecting phase transition and permeability changes induced by gemini surfactants on model vesicles. The effect of spacer and tail length on the morphology of model vesicles was also addressed by ^31^P NMR [25]. Insight on the interaction scheme was concomitantly provided by standard MD simulations carried out on a fully hydrated bilayer interacting with selected gemini surfactant molecules.

## Materials and Methods

### Materials

Cationic gemini surfactants, dimethylene bis (alkyldimethylammonium bromide), 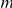-2-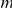 for 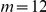, 14, and 18, and alkylene bis (dodecyldimethylammonium bromide), 12-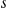-12 for 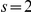, 6 and 10, were synthesized and purified according to standard methods [26]–[28]. The high purity of the products was ascertained by NMR, elemental analysis, surface tension and differential scanning calorimetry.

### Citotoxicity

#### Cell culture

NCTC 2544 cells (human skin keratinocyte cell line) [29], [30] were maintained in culture at 37°C, under 5% CO2, in RPMI-1640 medium (Sigma, St. Louis, MO) supplemented with 10% (v/v) heat-inactivated fetal bovine serum (FBS) (Sigma, St. Louis, MO), penicillin (100 U/mL) and streptomycin (100 *µg/mL). NCTC 2544 cells grow in monolayer and were detached by treatment with a trypsin solution (0.25%) (Sigma, St. Louis, MO).*

#### Cell viability assay

The cytotoxicity of gemini surfactants was evaluated in NCTC 2544 cells by a modified Alamar blue assay [23]. This method measures the redox capacity of the cells due to the production of metabolites as a result of cell growth and allows the determination of viability over the culture period without the detachment of adherent cells. The procedure was optimized in order to (i) obtain conditions similar to those found when applying transdermal patches, i.e. assessment of cell viability 24 h after exposure and 48 h after removing the external agent, (ii) identify cytotoxicity concentration limits, as well as discriminate the various surfactants in terms of the respective cytotoxicity.

For this study, gemini surfactants 12-2-12, 12-6-12, 12-10-12 and 14-2-14 were used, and each one tested for the 1, 5, 10, 25 and 50 mM concentrations. Moreover, the corresponding single tail surfactant DTAB was also employed for comparison. For each surfactant concentration, five independent assays, each repeated three times, were performed. Briefly, concentrated solutions of surfactant were prepared by dissolution in the culture medium (RPMI-1640), followed by filtration-sterilization using 0.22 *µ*m pore-diameter filters (Schleicher & Schuell, Dassel, Germany).

Twenty four hours before incubation with surfactants, a cell suspension was prepared in RPMI-1640 medium and 80×10^3^ cells/well were seeded in 48-well culture plate. Cells were incubated with the different surfactants, at the various concentrations, for 24 h under culture conditions (37°C and 5%CO_2_). After that, 0.3 mL of 10% (v/v) resazurin dye in RPMI-1640 culture medium was added to each well and, after 1 h of incubation at 37°C, 180 *µ*L of the supernatant were collected from each well and transferred to 96-well plates. The absorbance at 570 and 600 nm was measured in a SPECTRAmax PLUS 384 spectrophotometer (Molecular Devices, Union City, CA) and cell viability (as a percentage of control cells) calculated according to
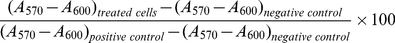
(1)


The positive control corresponds to cells not treated with surfactant, while the negative control corresponds to the same dilutions of the resazurin dye that was not incubated with cells.

Regarding the recovery assay, cells were maintained in RPMI-1640 medium free of surfactant, under culture conditions (37°C and 5%CO2), during a new 48 h period and then submitted again to the referred cell viability assay.

### Leakage

Large unilamellar vesicles (LUV) were prepared from chloroform stock solution of L-*α*-phosphatidylcholine (Chicken Egg PC, 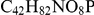, average MW: 770.123), L-*α*-phosphatidylethanolamine (Chicken Egg PE, 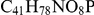, average MW: 746.608), L-*α*-phosphatidylserine (Porcine Brain PS, 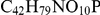, average MW: 824.966) and cholesterol (Chol, MW: 386.650). Since the phospholipids (Avanti Polar Lipids, Alabaster, AL) were obtained from natural sources, there is a mixture of hydrocarbon chain length, but acyl chains are mostly composed by 18 carbons, as established from the molecular formula and average weight of the components.

Briefly, lipids were mixed at the 1∶1∶1∶1 (PC:PE:PS:Chol) molar ratio and dried under vacuum in a rotator evaporator. The dried lipid film was hydrated with 80 mM calcein (Sigma, St. Louis, MO) in 50 mM HEPES and 1 mM EDTA (pH 8.4), submitted to 3 minutes of sonication, and then extruded 21 times through two stacked polycarbonate membranes of 100 nm pore diameter, using a Liposofast device (Avestin, Toronto, Canada), to obtain large unilamellar liposomes (LUV). Free calcein was separated from the dye-containing LUV by size exclusion chromatography on a Sephadex G-75 column, using a buffer (20 mM HEPES, 140 mM NaCl and 1 mM EDTA (pH 7.4)) with the same osmolarity than the calcein solution. Phospholipid concentration was measured by the Fiske and Subbarow method[31].

Liposome leakage assays were performed on a 96-well opaque plate, using a liposome concentration of 10 mM and the different surfactants (DTAB, 12-2-12, 12-10-12 and 14-2-14) in the concentrations of 5, 25 and 50 mM. The kinetics of leakage of calcein was followed at 37°C, for 20 minutes, in a SPECTRAmax Gemini EM fluorimeter (Molecular Devices, Union City, CA), using the excitation and emission wavelengths of 490 and 520 nm, respectively. The percentage of leakage was calculated according to
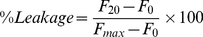
(2)


where 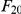 corresponds to the observed fluorescence after 20 minutes of incubation for the liposomes in the presence of surfactants, 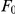 corresponds to fluorescence measured before the surfactants addiction (liposomes in the absence of surfactants), and 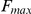 corresponds to the maximum fluorescence, which was obtained after the complete lysis of liposomes with 0.5% (v/v) Triton X-100. For each surfactant concentration, two independent assays (each repeated three times) were performed.

### DSC

Mixed liposomes were prepared according to a standard procedure derived from the original solvent evaporation method [32]. Briefly, lipids (Avanti Polar Lipids, Alabaster, AL) and surfactants (12-2-12, 12-10-12, 18-2-18) were dissolved in a chloroform/methanol mixture and dried under reduced pressure to form a homogeneous thin lipid film. Subsequently, by slowly agitating the solution inside a bath set to ca. 55°C, the resulting lipid film was resuspended in 10 mM tris-maleate, 50 mM KCl (pH 7) buffer to give the desired final lipid concentration. The final solutions were then subjected to vigorous vortexing conditions and left to equilibrate overnight inside a bath at a temperature slightly above the melting temperature of DPPC. This approach has been reported [6] as adequate to achieve a correct dynamical equilibrium.

DSC was performed on a Perkin Elmer Pyris 1. Volumes of 20 *µ*L of the liposomal suspension containing an average of 2 mg of DPPC:Chol were sealed in 50 *µ*L aluminium pans. An empty pan was used as reference [33], [34], so as to circumvent frequent seal breaking induced by vapor pressure created at higher temperatures, when water-filled pans are used. The samples were analyzed by heating-cooling cycles at scanning rates of 10°C/min over the temperature range 10-60°C. For data acquisition and subsequent analysis of thermograms, the software provided by *Perkin-Elmer* was used.

### ^31^P-NMR

DPPC liposomes were prepared by the solvent evaporation method, as previously described for the DSC studies. However, in this case, deuterated water was used to hydrate the lipid film and prepare the liposomes. The 12-2-12, 12-10-12 and 18-2-18 gemini, as well as the single tail DTAB, were included in this part of the work.

^31^P NMR spectra were acquired on a Varian Spectrometer, Unity-500 MHz, using a broadband probe. Typical acquisition parameters consisted of a 90° radiofrequency pulses, 40000 Hz spectral width and waltz broadband proton decoupling. The dwell time of 50 *µ*s, and 2000-4000 transients were accumulated for each free induction decay (FID) with a 3 s relaxation delay. The ^31^P chemical shifts were referenced externally to 85% 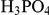 (0 ppm). Samples were allowed to equilibrate at least 30 min at a given temperature before the NMR signal was acquired at 30, 40 and 50°C, according to a standard procedure [25].

### Molecular Dynamics simulation

#### Systems

Following previous work[6], a set of molecular dynamics performed on the 12-2-12, 12-10-12, 14-2-14 and 18-2-18 gemini surfactants embedded in a DPPC bilayer was analyzed. A fully hydrated (1672 SPC water molecules) DPPC bilayer was generated and equilibrated before the insertion of the surfactant molecules. A relatively small bilayer, consisting of 72 phospholipid molecules equally distributed by the two leaflets was built by placing, in a regular grid, molecules with random rotation around the major axis. It should be noted that the size of the bilayer was large enough to accommodate a single embedded surfactant molecule, and allow the study of the respective interaction with the surrounding lipids.

Electroneutrality of the overall systems was imposed by adding a number of chloride ions corresponding to the total positive charge. It should be noted that, for simplicity, chloride ions were used instead of bromide because the latter are not implemented in the original force field. However, qualitative aspects related to the structure and dynamics are not expected to be significantly influenced, as extracted from similar systems [35].

In order to attain equilibrated systems within the timescale of the MD, surfactants were directly incorporated in a pre-equilibrated and fully hydrated DPPC bilayer by substituting one phospholipid molecule of the previous equilibrated bilayer [6].

#### Parameters and data analysis

All MD simulations were carried out in the NpT ensemble and under periodic boundary conditions, using the GROMACS package, version 3.3.3[36]–[38], and the GROMOS 96 53a6 force field [39], [40]. A standard time step of 2 fs was used for both the equilibration and production runs. Non-bonded interactions were computed on the basis of a neighbor list, updated every 10 steps. Long-range electrostatics were computed using the particle mesh Ewald (PME) method, as recommended for charged membrane simulations [41]. For Lennard-Jones energies, a cut-off of 1.4 nm was applied. Temperature and pressure were coupled to the Berendsen external baths maintained at 325 K (ca. 10 K above the gel to liquid crystalline phase transition of DPPC) and 1 bar (semi-isotropic pressure coupling, separately applied to the 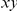 plane and 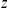 direction), with coupling constants of 0.1 and 0.5 ps, respectively.

To obtain a starting configuration, each system was firstly subjected to an energy minimization step. The system were then left to evolve up to 100 ns, using the LINCS algorithm [42] to keep bonds containing H atoms under positional restraint conditions. The first 40 ns were considered sufficient to attain equilibrated systems (converged value of the area per lipid), while the last 60 ns of production runs were subsequently subjected to standard analysis, such as atom-atom (group-group) distance distributions and radial distribution functions (rdf). MD trajectories were visualized, and configuration images extracted using the VMD 1.8.6 software [43].

## Results and Discussion

Very few chemical enhancers for transdermal drug delivery have been approved for clinical use, due to lack of efficiency or toxicity concerns. This work compaginates a detailed biophysical characterization of lipid bilayer-gemini systems with a cytotoxicity evaluation of the gemini surfactants in a skin irritation model. An integrated interpretation of the system dynamics and membrane disruption activity is provided in terms of gemini architecture.

### Cytotoxicity

Since cationic surfactants are known to disturb membrane integrity[4], [6], it is important to determine the extent of damage caused by cationic gemini surfactants.

In what follows, the cytotoxicity of several dicationic gemini surfactants (12-2-12, 12-6-12, 12-10-12, 14-2-14) was studied, and compared with a commercial single tail surfactant (DTAB). These surfactants, selected from previous studies [6], [17], were deemed sufficient to account for variations in the spacer and tail length. It should be noted that longer tail gemini surfactants, 18-2-18, were not included in this part of the work due to the low solubility in the culture medium solution. The NCTC 2544 cell line, a human skin keratinocyte cell line, was chosen as a model of skin irritation, while an Alamar blue assay was used to assess the respective cell viability.

**Figure 1:**
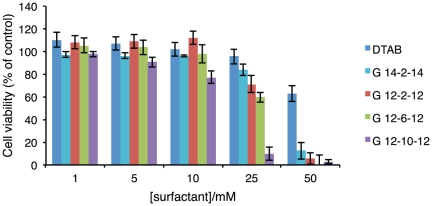
Viability of NCTC 2544 cells after 24 h of surfactant exposure. Cell viability values are presented as mean±SD of a characteristic profile (3 repetitions) selected from 5 independent experiments.

Results for 24 h of exposure are presented in terms of cell viability, as a percentage of control cells, and depicted in Figure 1. A first observation indicates that, for the lower concentrations tested (up to 10 mM), none of the surfactants reveals a significant cytotoxicity upon the cellular line. However, from 25 mM onwards, a strong toxicity is observed for some of them. Regarding the latter concentration results, from which the cytotoxicity trend becomes distinguishable for the different surfactants, it is clear that toxicity is higher for the gemini than for the corresponding single tail surfactant. Also clear from this representation, is that toxicity increases as the spacer length increases and that longer tail surfactants are less toxic than shorter ones.

Although results point to a higher cytotoxicity of gemini surfactants than for the corresponding single tail surfactant, it is possible that an important permeation enhancement occurs for gemini concentrations below the cytotoxicity limits. In fact, as will be discussed later, in the majority of the cases gemini surfactants are more effective in disrupting the membrane than the single-tail counterpart. This means that a low amount of gemini may be needed to achieve the same effect of a significantly higher amount of DTAB.

Attempts to relate the structure of alkylammonium bromide surfactants and the respective cytotoxicity effects, have been previously made. It has been shown that the cytotoxicity of surfactants decreases as the alkyl chain length increases [44], [45], in a trend compatible with the current observations. It has been also reported that more hydrophilic surfactants (larger head groups) present a significantly lower cytotoxicity [46]. A relation between the surfactant structure and the microbicide and contraceptive properties has been presented [4] for the single-tailed quaternary ammonium surfactants. In this case, the absolute concentration of surfactant (controlled by the CMC), is considered crucial for the respective effect. Results reported suggest that quaternary ammonium surfactants interact differently with different types of cells. Beyond the alkyl chain length dependence, the presence of specific polar heads and some counterions are also expected to contribute for the global toxicity [4], [46]. However, as far as polar heads and counterions are concerned, it is possible to replace them by more biocompatible ones [19], [21], [47], [48].

**Figure 2:**
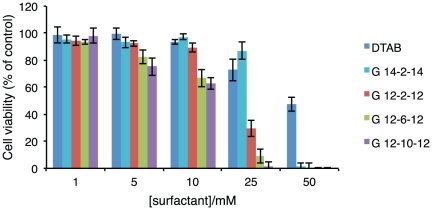
Recovery capability of NCTC 2544 cells 48 h after removing surfactants from the culture medium. Cell viability values are presented as mean±SD of a characteristic profile (3 repetitions) selected from 5 independent experiments.

Aiming to mimic the recovery process of skin cells after removing an actual transdermal device, cell viability was assessed 48 h after surfactant remotion. Results, as presented in Figure 2, indicate that for a surfactant concentration higher than 25 mM, the toxic effect is irreversible, i.e. cell viability does not increase 48 h after removing the surfactant from the culture medium. The cytotoxicity trend previously discussed is even more clear from this representation.

### Membrane integrity

In order to correlate the cytotoxicity effect of surfactants with the respective ability to induce cell membrane destabilization, a liposome leakage assay was performed using liposomes containing calcein, with a composition that mimic cell membrane, and surfactants in the range concentrations used for the cytotoxicity study.

**Figure 3:**
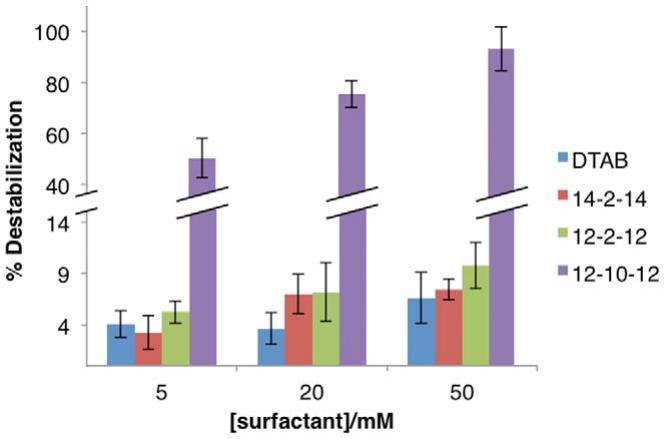
Membrane destabilization (%± SD) as obtained by the calcein fluorescent die released from PE:PC:PS:CHO (1∶1∶1∶1) liposomes, for the indicated surfactant concentrations. Experiments were conducted at room temperature.

As shown in Figure 3, it is clearly observed that gemini surfactants are generally more effective in promoting membrane destabilization than DTAB. Following an order of increasing effect, one has the 14-2-14, 12-2-12 and 12-10-12 gemini, with the latter placed drastically above the others. These observations suggest that longer tail gemini surfactants are less disruptive, but that the major effect is associated to the spacer length. For the range of concentrations under study, the longest hydrophobic spacer promotes the most significant liposome disruption effect, which is directly correlated with the higher cytotoxicity effect induced by this surfactant. These results indicate that most probably the cytotoxicity promoted by these compounds is, at least, partially due to their capacity to induce cell membrane destabilization.

### Thermotropic behavior

The impact of cholesterol on biological membranes has been extensively studied both experimentally [49] and theoretically[50]–[52]. Moreover, cholesterol is one of the major components of the SC and plays an important role in the barrier function. Therefore, the inclusion of cholesterol in the composition of liposomes to model the skin barrier function has been frequently used to study skin permeation enhancers [53], [54].

A well characterized model of DPPC:Chol was used to evaluate the effect of gemini surfactants on the respective thermotropic behavior. Three gemini surfactants (12-2-12, 12-10-12 and 18-2-18) were used in this part of the work. The fraction of cholesterol in the lipid composition was supported on a preliminary study, where an increasing amount of cholesterol (up to 50%) was incorporated in a DPPC:Chol mixture (data not presented).

Regarding DPPC bilayers, a heating scan induces a transition from a highly organized state (gel phase), in which molecular motions are severely restricted and the alkyl chains of lipids are in a all-*trans* conformation, to a state of higher molecular mobility and conformation disorder (fluid phase), in which the alkyl chains present *gauche* defects[55]. The presence of cholesterol produces a progressive decrease in the temperature of the main phase transition characteristic of DPPC, which is replaced by a new asymmetric endothermic event. This has been ascribed to the overlapping of two symmetric peaks [56], [57]. A cooperative transition, detected by a sharp component originally centered at 40.2°C, is shifted to lower values of temperature as the concentration of cholesterol increases. In turn, a broad component, centered at a slightly higher temperature, is shifted towards higher temperatures as the concentration of cholesterol increases, becoming undetectable for a cholesterol concentration of ca. 50 mol% [58]. The transition energy of the broad component also increases as Chol concentration increases, reaching a maximum when the cooperative component vanishes, i.e. close to 20 mol%[58], [59]. The existence of two components in the main transition for Chol concentrations up to 20 mol% suggests phase separation in the membrane plane, i.e. coexistence of DPPC domains with a small amount or no cholesterol (sharp transition) and cholesterol-rich DPPC domains (broad transition). From the molecular point of view, it has been suggested that cholesterol molecules are predominantly located in the hydrophobic region of the bilayer [52]. After occupying the free volume available, that in fact is known to be higher in the bilayer centre, a consequent lowering of the conformational freedom of the alkyl chains occurs. This induces an increase in the lateral area of the membrane that, in turn, results in some increase of the free volume at the surface. When the fraction of cholesterol reaches a critical fraction of 5 mol%, cholesterol molecules can move to the lipid/water interface, compatible with the formation of a new dynamical structure, denoted as liquid-ordered phase [60], with biological relevance.

Thus, an amount of 15 mol% of Chol was considered enough to study the effect of gemini in a lipid model stabilized by the presence of cholesterol, without completely affecting the original phase transition of DPPC model, which is convenient to assess the gemini influence.

**Figure 4:**
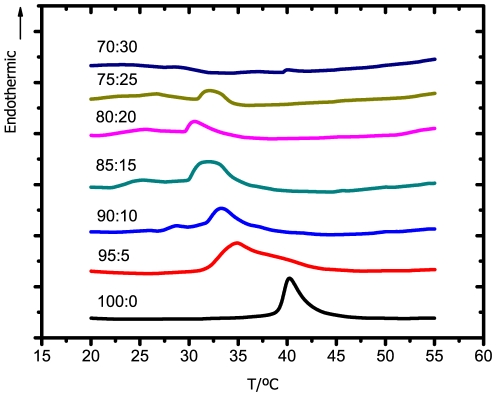
DSC thermograms of the DPPC:Chol:12-10-12 system for the lipid:surfactant molar ratios indicated. A scanning rate of 10°C/min was used.

Thermograms obtained for DPPC:Chol:12-10-12 system, as a function of 12-10-12 gemini concentration, are presented in Figure 4, while values of 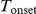 and 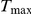 are summarized in Table 1. The addition of 5% of 12-10-12 surfactant promotes a significant broadening of the original transition peak, as well as a marked decrease of the transition temperature. Increasing the concentration of gemini up to 15 mol% slightly shifts the transition temperature to even lower values. After that, for 20 and 25 mol%, the addition of gemini seems not to significantly increase the effect upon the model. However, for 30 mol% of gemini, the transition completely vanishes. These results are an indication of the ability of the 12-10-12 surfactant to lower the order of the model membrane, even at a low gemini concentration. Such disordering effect is compatible with a decrease of the barrier function and, consequently, an increase of the respective permeation across the membrane may be expected.

**Table 1:** Data extracted from the DSC thermograms.

lipid:surfactant	12-10-12		12-2-12		18-2-18	
100∶0	38.9±0.2	40.0±0.3	40.0±0.2	41.4±0.4	41±1	42±1
95∶5	31.8±0.1	35.0±0.2	39±2	41±2	42±1	46±2
90∶10	31.0±0.3	34±2	35.1±0.8	40±1	43.1±0.1	46.6±0.2
85∶15	30±1	32.5±0.6	28.0±0.8	37.3±0.1	43.8±0.6	47.4±0.1
80∶20	25±4	28±3	28.9±0.4	31.8±0.1	45.05±0.6	47.6±0.1
75∶25	27±5	29±4	28.2±0.3	30.9±0.3	48±1	50±1
70∶30	-	-	28.5±0.1	31.0±0.2	46±1	49±1

Values of 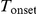 (left columns) and 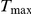 (right columns), indicated by mean ±SD (minimum of three repetitions), for the detected phase transition, extracted from the DSC thermograms of DPPC/CHO systems for the indicated molar percentages. A scanning rate of 10°C/min was used.

**Figure 5:**
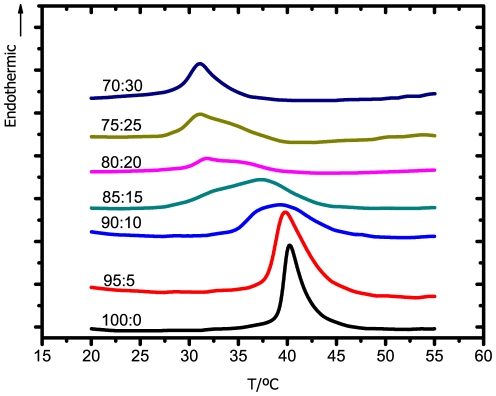
DSC thermograms of the DPPC:Chol:12-2-12 system for the lipid:surfactant molar ratios indicated. A scanning rate of 10°C/min was used.

Regarding the 12-2-12 surfactant, thermograms are presented in Figure 5, while the values of the respective transition temperatures are shown in Table 1. Although the trend is similar to that obtained for the longer spacer surfactant, the effect for the smallest concentration of surfactant is comparatively small. As the concentration of gemini increases, a progressive decrease in the transition temperature and the broadening of the respective peak are clearly visible. In comparison to the 12-10-12, this shorter spacer surfactant promotes a broader phase transition, but not the respective disappearance, even at the highest concentration.

**Figure 6:**
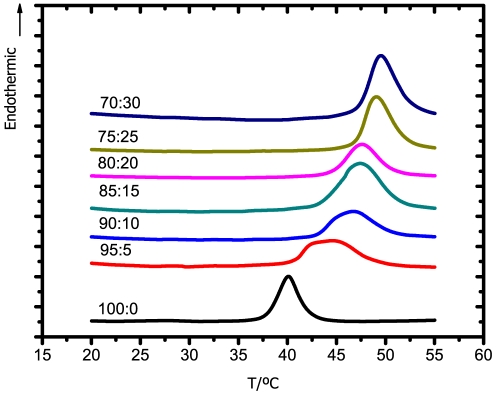
DSC thermograms of the DPPC:Chol:18-2-18 system for the lipid:surfactant molar ratios indicated. A scanning rate of 10°C/min was used.

The 18-2-18 surfactant, in turn, was responsible to induce an opposite effect. As observed in Figure 6, instead of the transition peak shifting to lower temperatures, this surfactant promotes the increase in the transition temperature. Furthermore, in the presence of 30 mol% of this longer tail surfactant, the transition temperature of the DPPC:Chol model is higher than for the DPPC model in the absence of cholesterol. These observations indicate that the longer tail gemini surfactant induces the formation of more ordered structures.

To summarize, surfactants containing shorter tails (12-10-12 and 12-2-12) promote a decrease in the overall order of the bilayer, while the opposite effect was found for longer tail surfactant (18-2-18). Also observed is that, among the shorter tail surfactants, the one with longer spacer (12-10-12) was responsible for a more pronounced disrupting effect upon the model membrane, in accordance with the previous discussed results.

Comparing these results for the DPPC:Chol:gemini systems with those previous obtained for the DPPC:gemini systems [6], reveals similar trends. As such, the simpler DPPC model was chosen in the subsequent NMR and MD studies.

### Morphology

In order to check morphological changes induced by gemini surfactants in a model of lipid membrane, ^31^P-NMR spectra were acquired for DPPC liposomes in the presence of DTAB, 12-2-12, 12-10-12, 18-2-18 at three different temperatures (30, 40 and 50°C). Monitoring the ^31^P heteronucleus present in the phosphatidic group of DPPC, it is possible to follow changes in the overall membrane structure [25].

**Figure 7:**
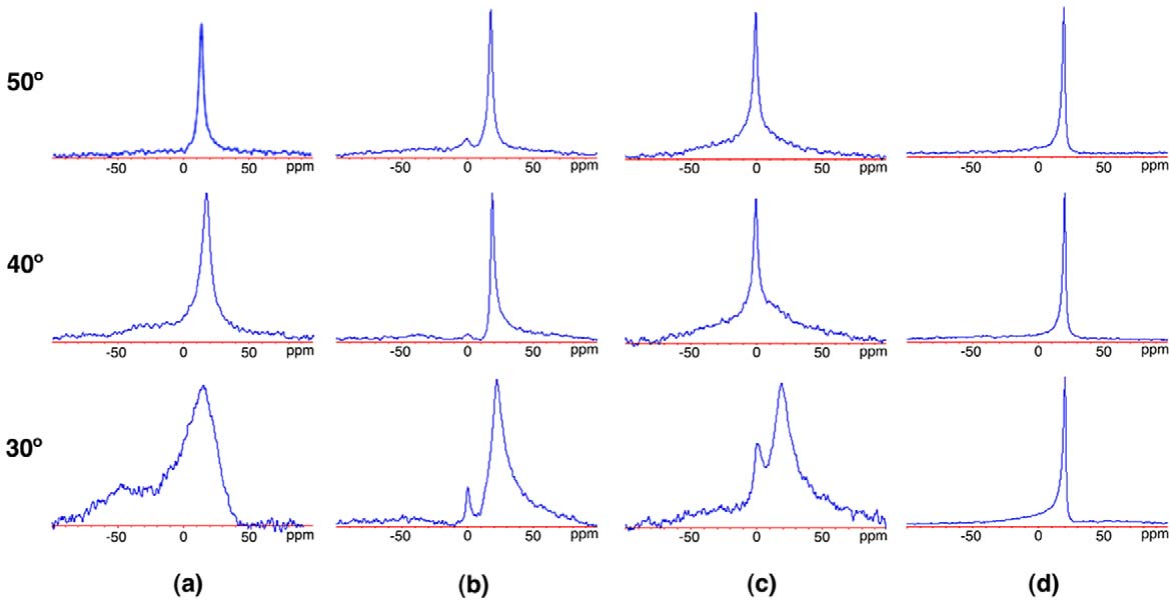
^31^P-NMR spectra of a DPPC:surfactant systems ([DPPC] = 30 mg/mL, 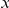
(surfactant) = 20 mol%), in the (a) absence of surfactant, and in the presence of (b) DTAB, (c) 12-2-12 and (d) 12-10-12. The chemical shift is represented in the horizontal axis.

Spectra obtained for the neat DPPC liposomes are presented in panel (a) of Figure 7. The shape of the peak observed at *T* = 30°C is characteristic of a lipid bilayer in the gel phase [61], [62]. As the temperature increases, the peak becomes sharper and the asymmetric shoulder vanishes. This new shape indicates that the dynamic behavior of the membrane is now characteristic of a fluid-like phase.

As depicted in panel (b), the incorporation of DTAB also promotes some degree of fluidization, now for lower temperatures. Moreover, there is a visible part of the phosphorus detected at zero chemical shift. This isotropic behavior may be due to a partial micellization of original liposome structures, or to conversion into small vesicles. The bilayer persists, however, even at the highest temperature (50°C).

This effect is even more visible in the presence of the 12-2-12 surfactant, as depicted in panel (c) of the same Figure. Now, the fraction of phosphorus presenting an isotropic behavior, coexisting with the bilayer structure, is higher from the lowest temperature (30°C) onwards, and a complete conversion is observed for the higher temperatures, 40 and 50°C. Since the solution is more viscous and clearer than the corresponding neat DPPC and DPPC:DTAB ones, a cubic arrangement is a plausible guess. In fact, the broadening of the basis of the peak and the respective symmetry, more marked for the highest temperature, is compatible with such a structural arrangement [63].

The gemini surfactant with increased spacer length, 12-10-12, shows a very distinct effect, panel (d). In this case, the presence of a longer spacer surfactant promotes from the lowest temperature (30°C) a dynamic behavior characteristic of a lipid bilayer in the fluid phase, observed in the neat DPPC system only for the highest temperature. However, there is no evidence of any micellization or dissolution of the bilayer structure. In this case, the solution was slightly opaque with a viscosity similar to those of DPPC.

The longer tail surfactant, 18-2-18, was very difficult to evaluate through this method, as a consequence of the occurrence of phase separation, which is compatible with the more ordered structure suggested by the other techniques (spectra not presented). This is confirmed by the white colored, markedly opaque appearence of the solution, in contrast to that of the neat DPPC solution.

### Molecular insight

The MD study focused on the 12-10-12, 12-2-12, 14-2-14 and 18-2-18 gemini surfactants, deemed sufficient for a description of the variations induced by spacer and tail lengths. This section follows previous work by the authors [6], [35], in which the 12-2-12, 12-10-12 and the 18-2-18 gemini molecules inserted in DPPC and DODAB bilayers were studied by molecular dynamics, and general trends established. In the present work, we provide a detailed quantitative description of the gemini conformation, positioning within the bilayer and behavior towards the solvent. A new system, comprising DPPC and the 14-2-14 gemini, was also included in this study to allow a more direct comparison with the cytotoxicity results, in which the 18-2-18 gemini was not used due to insolubility at the relevant concentration range.

**Figure 8:**
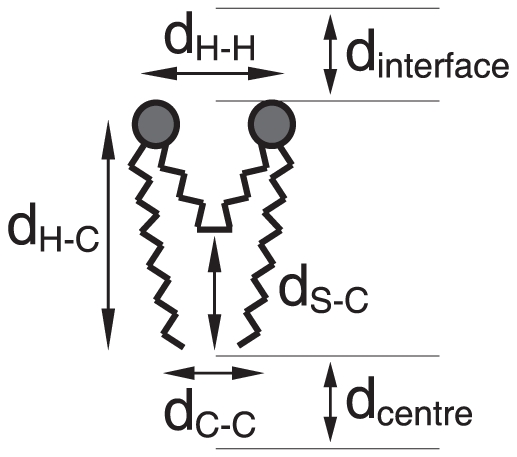
Schema of the distances, extracted from the MD, used to characterize the conformation and relative position of gemini molecules in the bilayer. A summary of the respective values for the 12-2-12, 12-10-12, 14-2-14, and 18-2-18 surfactants are presented in Table 2.

Table 2 summarizes some characteristic distances found in the gemini molecules, and respective positioning relative to the water interface and bilayer center (see Figure 8 for a schema of the respective distances). For the 12-2-12, 14-2-14 and 18-2-18 surfactants, it is seen that the head-head distance (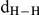) is highly controlled by the short spacer, that display a common figure of 0.5 nm. The longer spacer surfactant, 12-10-12, presents a significantly larger 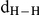, but much shorter than that expected from a fully extended 10-carbon chain. This suggests that, in this case, the spacer bends. As the distance from the central carbons of the spacer to the terminal carbon in the tail (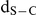) is considerably smaller than the corresponding head-tail distance, it can be concluded that the spacer bends towards the interior of the membrane, as previously suggested [6]. Naturally, no such bending is possible with the 2-carbon spacers.

**Table 2:** Data extracted from the MD simulations.

	12-10-12	12-2-12	14-2-14	18-2-18
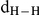 /nm	1.4±0.2	0.5±0.1	0.5±0.1	0.5±0.1
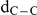 /nm	1.9±0.7	1.0±0.6	0.9±0.4	0.9±0.4
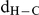 /nm	1.2±0.1	1.2±0.1	1.3±0.1	1.7±0.1
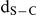 /nm	0.9±0.3	1.2±0.1	1.3±0.2	1.7±0.2
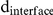 /nm	0.49±0.07	0.21±0.02	0.20±0.04	0.09±0.03
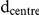 /nm	0.21±0.08	0.36±0.04	0.32±0.05	-0.12±0.04

General data on the average conformation (top of the table) and relative positioning (bottom of the table) of the indicated gemini molecules inserted in a fully hydrated DPPC bilayer as extracted from the MD simulation at 325 K. See Figure 8 for a schema of the respective distances.

In the case of the head-tail distance (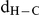), it is approximately the same in the 12-2-12, 12-10-12 and 14-2-14 surfactants, and higher for the 18-2-18 one, as expected. In turn, a longer spacer promotes a longer distance between the terminal methyl groups (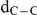). Note that the 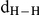 for the 2-carbon spacers is roughly one third of that corresponding to the 10-carbon counterpart, while the distance between the terminal carbons is one half. The latter is the quantity in which a larger fluctuation is observed, as expected from the commonly observed lower order in the central region of the bilayer.

The analysis of preferential vertical positioning of gemini molecules embedded in the lipid bilayer relative to the water interface and bilayer centre also show important differences. Heads of the longer spacer surfactant (12-10-12) are fully embedded in the membrane, at 0.49 nm from the water interface, while the corresponding shorter spacer molecule (12-2-12) is fixed in a higher positioning, closer to the interface but yet embedded. In both cases, the terminal methyl groups of the tails do not reach the interleaflet bilayer, with 12-2-12 farthest positioned from the core. In turn, polar heads of the longer tail surfactant (18-2-18) are almost leveled with the phospholipid polar heads, while the terminal methyl groups of the tails slightly interdigitate with the opposite leaflet. The 14-2-14 gemini behaves as an intermediate case, with the terminal methyl groups reaching further towards the centre, but not attaining the central part of the bilayer, as the longest 18-2-18 surfactant.

**Figure 9:**
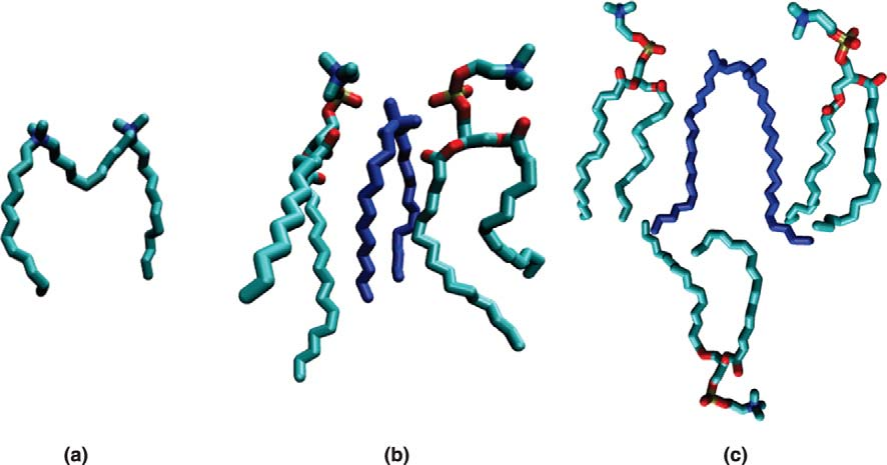
Snapshots selected from the MD simulations, illustrating (a) a typical conformation of the 12-10-12 molecule (the long hydrophobic spacer bending towards the interior of the bilayer), and the positioning of the (b) 12-2-12 and (c) 18-2-18 molecules relative to the neighboring phospholipid molecules. The larger conformational freedom found close to bilayer centre observed for the short tail surfactant contrasts with the interdigitation evidence observed for the long tail surfactant.

**Figure 10:**
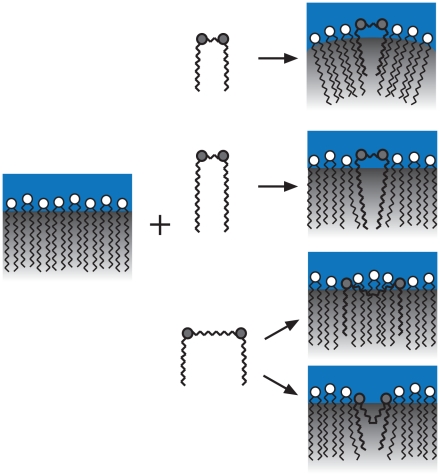
Pictorial illustration of the positioning and general conformational of gemini molecules embedded in the membrane, as well as the consequent morphological modification of the latter, based on the ^31^P-NMR and MD simulation results. From the top to the bottom, gemini surfactants represented correspond to short spacer/short tail, short spacer/long tail and long spacer/short tail architectures.

These results confirm, as already suggested [6], [35], that the interaction behavior of gemini surfactants and model membranes is dependent on the length of both the spacer and tails. The described low vertical positioning of the 12-10-12 molecule relative to the bilayer interface can be explained by some effort of the system in order to reduce the contact of the hydrophobic spacer with the polar heads of the phospholipids and water. In fact, when inserted in the bilayer, the spacer bends towards the respective interior, as seen in the panel (a) of Figure 9, in which a typical conformation of the molecule, as extracted from the MD simulation, is presented. A similar behavior was observed for the 12-2-12 molecule. However, the smaller hydrophobic spacer allows a higher positioning of the polar heads that, consequently, promotes the formation of a lower density region close to the bilayer centre (see panel (b) of Figure 9). This effect, that is comparatively smaller for the previous longer spacer surfactant, is compatible with the disordering effect found in the thermotropic and permeability characterization, extracted from DSC and leakage assays. Furthermore, the micellization effect observed for the 12-2-12 surfactant in the morphological studies is expected from the average conformation and relative vertical positioning adopted by the 12-2-12 molecule when embedded in the membrane. A strong reduction of density close to the bilayer interleaflet suggests an increase of the curvature, compatible with micelle formation. In the case of the 18-2-18 surfactant, no evidence of disruption is attained from the simulation results. This molecule adopts a conformation and a relative vertical positioning similar to the phospholipids molecules. Moreover, a slight interdigitation (see panel (c) of Figure 9) can explain some increase of order suggested by the DSC and morphological studies. The 14-2-14 surfactant behaves as an intermediate case of the 12-2-12 and 18-2-18, as previously remarked. An illustrative scheme of the gemini-membrane interaction, as a function of tail and spacer length, is depicted in Figure 10.

**Figure 11:**
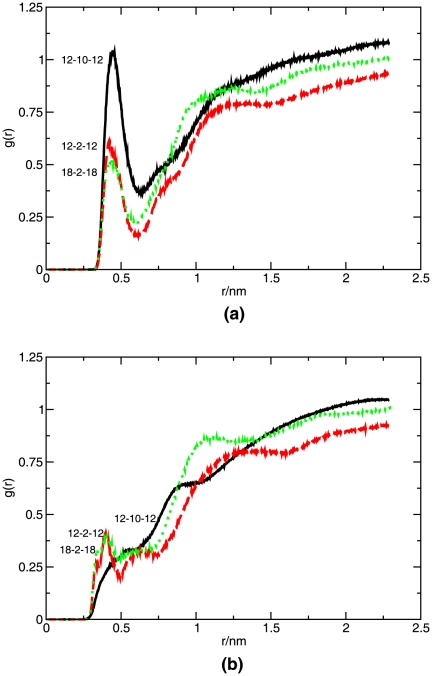
Radial distribution functions of water relative to the (a) gemini polar heads, and (b) gemini spacer, calculated from the MD. Simulations were carried out at 325 K.

A further inspection, provided by rdf analyses, was made on the water accessing the spacer and heads of the gemini molecules. As extracted from panel (a) of Figure 11, the 12-10-12 gemini molecule presents a higher amount of water in the proximity of the respective polar heads. This molecule was found more deeply inserted in the membrane, with the polar heads further from the water interface, which could suggest the opposite result. However, due to a strong local disturbance of the membrane, promoted by the presence of a long hydrophobic spacer almost leveled with the polar heads of the phospholipids, the penetration of water is expected to increase. In contrast, a significant smaller amount of water was found in the vicinity of the polar heads for both shorter spacer surfactants, 12-2-12 and 18-2-18. This observation may, along the same lines, be ascribed to the smaller disruption of the membrane order at the level of the polar heads. As visible in panel (b) of the same Figure, the contact of the spacers with water is significantly smaller than that of the polar heads, and the trend less trivial. These distributions seem to reflect the vertical positioning of the spacer relative to the water-membrane interface, rather than the order in the membrane. The bending conformation of the long spacer makes the 12-10-12 molecule less accessible to water. In the case of the short spacer surfactants, 12-2-12 and 18-2-18, water is found for a smaller radius, with the longer tail surfactant presenting a peak for the smallest radius. Results pertaining to the 14-2-14 surfactant were very coincident with those obtained for the short spacer surfactants and, for clarity, were omitted in the rdf representations. These observations are compatible with the previous discussion.

In summary, no significancet toxicity was found on the NCTC 2544 cell line for any of the surfactants, in a concentration range up to 10 mM. From 25 mM onwards, a clear trend indicates that toxicity is higher for the gemini surfactants than for the corresponding single-tailed surfactant. In terms of surfactant structure, cytotoxicity increases as the spacer length increases, and decreases with the increase of tail length. Recovery tests also indicate that for surfactant concentrations higher than 25 mM the cytotoxicity effect is irreversible.

Permeability of liposomes to calcein, in the presence of the same surfactants, show a similar trend. In this case, the 12-10-12 surfactant was responsible for a drastic loss of integrity from the membrane.

The cationic gemini surfactants under study are able to alter the thermotropic behavior of the DPPC:Chol model. Shorter tail surfactants, 12-2-12 and 12-10-12, reduce the temperature of the original phase transition, which is compatible with a decreasing membrane order. The opposite effect was found for the longer tail surfactant, 18-2-18. Regarding spacer length variation, no significant differences were observed. However, the longer spacer surfactant seems to be more effective in the disruption of the membrane for smaller concentrations.

The morphological study returns some clues about the perturbation mechanism behind gemini surfactants. It seems clear from the NMR results that shorter tail surfactants are much more active in terms of perturbation of the original DPPC bilayer structure than the corresponding longer tail surfactant. Relative to spacer length, it was suggested that a shorter spacer promotes an increase of the curvature of the bilayer structure, while a longer spacer seem to drastically disturb the order without destroying the original bilayer structure.

Molecular dynamics simulation supports the most important findings on the interaction between the cationic gemini surfactants with model membranes, and provides insight into the respective mechanism that governs the interaction between the cationic gemini surfactants, of variable spacer and tail length, with membranes. From the analysis of various systems, it was possible to establish a number of factors that contribute for the disrupting effect of lipid membranes. These factors include preferential conformation of surfactant molecules embedded in the bilayer structure and respective positioning relative to the bilayer centre. Such factors can be directly related to the chemical structure of surfactants.
